# Genome-wide identification of ATG genes and their expression profiles under biotic and abiotic stresses in *Fenneropenaeus chinensis*

**DOI:** 10.1186/s12864-024-10529-2

**Published:** 2024-06-20

**Authors:** Chenhui Guan, Yalun Li, Qiong Wang, Jiajia Wang, Caijuan Tian, Yuying He, Zhaoxia Li

**Affiliations:** 1 School of Marine Science and Engineering, Qingdao Agricultural University, Qingdao, 266237 PR China; 2 National Key Laboratory of Mariculture Biobreeding and Sustainable Goods, Yellow Sea Fisheries Research Institute, Chinese Academy of Fishery Sciences, Qingdao, 266071 PR China; 3 Function Laboratory for Marine Fisheries Science and Food Production Processes, Pilot National Laboratory for Marine Science and Technology, Qingdao, 266200 PR China; 4 Jiangsu Key Laboratory of Marine Bioresources and Environment, Jiangsu Key Laboratory of Marine Bio-technology, Jiangsu Ocean University, Lianyungang, Jiangsu 222005 PR China

**Keywords:** *Fenneropenaeus chinensis*, Autophagy, WSSV, Low-salt stress

## Abstract

**Background:**

Autophagy is a conserved catabolic process in eukaryotes that contributes to cell survival in response to multiple stresses and is important for organism fitness. Extensive research has shown that autophagy plays a pivotal role in both viral infection and replication processes. Despite the increasing research dedicated to autophagy, investigations into shrimp autophagy are relatively scarce.

**Results:**

Based on three different methods, a total of 20 members of the ATGs were identified from *F. chinensis*, all of which contained an autophagy domain. These genes were divided into 18 subfamilies based on their different C-terminal domains, and were found to be located on 16 chromosomes. Quantitative real-time PCR (qRT-PCR) results showed that ATG genes were extensively distributed in all the tested tissues, with the highest expression levels were detected in muscle and eyestalk. To clarify the comprehensive roles of ATG genes upon biotic and abiotic stresses, we examined their expression patterns. The expression levels of multiple ATGs showed an initial increase followed by a decrease, with the highest expression levels observed at 6 h and/or 24 h after WSSV injection. The expression levels of three genes (ATG1, ATG3, and ATG4B) gradually increased until 60 h after injection. Under low-salt conditions, 12 ATG genes were significantly induced, and their transcription abundance peaked at 96 h after treatment.

**Conclusions:**

These results suggested that ATG genes may have significant roles in responding to various environmental stressors. Overall, this study provides a thorough characterization and expression analysis of ATG genes in *F. chinensis*, laying a strong foundation for further functional studies and promising potential in innate immunity.

## Background

*Fenneropenaeus chinensis*, commonly known as Chinese shrimp, is a highly valued species in China’s aquaculture industry. Its distribution is mainly concentrated in the Yellow Sea and Bohai Sea of China, as well as the western and southern coast of the Korean Peninsula [1]. Due to the advancement of aquaculture techniques, *F. chinensis* emerged as the most important shrimp species for cultivation in China during the 1990s [2]. *F. chinensis* and other crustaceans often encounter changes in environmental factors, such as extreme temperatures, salinity, hypoxia and abnormal acid-base levels. Environmental factors can disrupt homeostatic equilibrium and cause the fluctuations in the neuroendocrine, physiological, and behavioral status of aquatic animals, negatively impacting their health [3]. Saline-alkaline water aquaculture is becoming a promising solution to accommodate the growing needs of the aquaculture industry. Therefore, it is important to investigate the physiological changes and molecular responses in aquatic animals adapting to this environment. *F. chinensis* is highly sensitive to changes in salinity levels, particularly in low-salt environments, which is considered a major stressor. It is essential to understand the effects of low salinity on *F. chinensis* to develop effective mitigation strategies in *F. chinensis* aquaculture. The sensitivity to low salinity is rooted in the osmoregulatory mechanisms employed by these organisms to maintain internal salt and water balance. The balance is crucial for their metabolic processes and impacts the reproductive and developmental processes of shrimps. In addition to abiotic stress, the significant environmental variations were accompanied by severe bacterial and viral infections, resulting in an epizootic breakout that caused great losses to the aquaculture industry [4]. White spot syndrome virus (WSSV) is a major pathogen in shrimp aquaculture. It is a double-stranded DNA virus that causes white spot disease [5–7]. The mortality rate of *F. chinensis* infected with WSSV can reach 100% within a week. WSSV first emerged in the late 20th century, causing huge economic losses. Despite extensive research, no effective solution to the WSSV problem has been conducted, and it remains one of the most fatal pathogens in *F. chinensis* aquaculture [8].

Autophagy is a conserved intracellular degradation system found in eukaryotes. In autophagy, an isolation membrane emerges suddenly in the cytoplasm, which then expands and transforms into double membrane-bound structure called an autophagosome [9–11]. During this process, a portion of cytoplasm, including proteins and organelles, is sequestered into the autophagosome. The autophagosome then fuses with a lysosome (or a vacuole, in yeast and plants). The inner membrane, referred to as an autophagic body in yeast, is exposed to lysosomal hydrolases and degraded along with its contents [9–11]. The primary function of autophagy is to maintain cellular homeostasis by recycling intracellular materials, which promotes stress resistance and longevity [11]. Autophagy has emerged as a crucial cellular process with implications for health and immunity in all eukaryotic organisms. Research has shown that a range of autophagy-related genes play important roles in both biotic and abiotic stresses in plants and animals, participating in sugar metabolism, antimicrobial peptide regulation, and other pathways [12, 13]. In yeast, there are over 36 autophagy-related genes (ATG) have been identified. Most of these genes have corresponding homologous genes in mammals [14, 15]. Drought and salt stress cause ion stress which induces oxidative damage in plant cells. Autophagy repairs this damage under the activation of various biological factors [16]. Silencing *OsATG2* and *OsATG7* inhibits autophagy and reduces wheat salt tolerance [17]. Overexpression *of MdATG18a* enhances the adaptability of apples to drought stress [18]. In animal studies, it has been found that the ATG5-ATG12 conjugate functions in the mouse innate antiviral immune system, enhancing their bactericidal activity [19]. For the banana shrimp, interference with either ATG3 or ATG6 during WSSV challenge resulted in a decrease in autophagic levels [20]. Therefore, autophagy-related research has recently gained increasing attention from the scientific community, but it is relatively uncommon in crustaceans. Given the growing importance of shrimp aquaculture and the risks posed by viral infections and environmental stress, it is crucial to comprehend the autophagy-related mechanisms in this economically vital species. However, the precise details of the *F. chinensis* autophagy-related gene family, its structure, and its specific roles in the response to WSSV infection and low-salt stress have not been comprehensively elucidated.

The objective of this study is to identify and characterize the ATG gene family in *F. chinensis* and investigate their expression patterns in response to biotic and abiotic stresses. The study aims to contribute to the advancement of shrimp aquaculture and the management of viral diseases and environmental factors that pose a threat to this industry.

## Results

### Identification and characterization of ATG genes in *F. chinensis*

In the *F. chinensis*, a total of 20 ATG genes were identified and named according to the rules for ATG gene nomenclature (Table 1). These ATG genes (designated as *FcATGs*) were classified into18 subfamilies, including *FcATG*1-3, *FcATG*4B, *FcATG*4D, *FcATG*5-8, *FcATG*8B, *FcATG*9-10, *FcATG*12-14, *FcVPS*15, *FcATG*16 -18, and *FcATG*101. The predicted molecular weight of *F. chinensis* ATG proteins were in the range of 13.63 kDa to 241.9 kDa and the deduced isoelectric points ranged from 4.57 to 9.69. The *FcATG*12 gene had the smallest molecular weight of 13.63 kDa, while the *FcATG*2 gene had the largest molecular weight of 241.9 kDa. The instability index of three ATG proteins, namely *FcATG*12, *FcATG*14, and *FcATG* 101, was less than 40, indicating their stability. On the other hand, the remaining 17 proteins were predicted to be unstable. All ATG proteins had GRAVY values less than 0, indicating their hydrophilic nature. The open reading frames (ORFs) of ATG genes ranged from 360 to 6,546 bp, with predicted protein lengths ranging from 119 to 2181 amino acids (aa). Subcellular location prediction revealed that 12 ATG proteins are located in the nucleus, while the remaining proteins are mainly present in the Golgi apparatus, cytoplasm, and mitochondrion. The cDNA sequences of these ATG genes have been submitted to the GenBank database, and their characteristics are summarized in Table 1.

**Table 1:** The characteristics of ATG proteins in *F. chinensis*

Protein name	ID	ORF length(bp)	Amino acids	Molecular weight	PI	Instability index	Aliphatic index	GRAVY	Subcellular localization
ATG1	LOC125025612	2517	838	89941.75	9.26	67.13	70.62	-0.442	Nucleus
ATG2	LOC125043934	6546	2181	241858.27	5.29	48.19	82.14	-0.339	Nucleus
ATG3	LOC125036151	951	316	35726.78	4.57	46.46	71.27	-0.614	Nucleus
ATG4B	LOC125047743	1233	410	47538.38	4.98	53.27	81.54	-0.288	Cytoplasm
ATG4D	LOC125033004	2259	752	83824.62	7.57	59.27	66.22	-0.560	Nucleus
ATG5	LOC125047653	810	269	31103.47	5.59	43.43	84.05	-0.407	Nucleus
ATG6	LOC125039134	1275	424	47999.00	5.08	41.28	81.91	-0.435	Nucleus
ATG7	LOC125046156	2091	696	76306.06	5.53	44.12	88.74	-0.112	Nucleus
ATG8	LOC125042574	360	119	14087.20	8.59	41.55	76.13	-0.584	Golgi apparatus
ATG8B	LOC125046396	369	122	14523.69	9.69	51.40	88.61	-0.577	Cytoplasm
ATG9A	LOC125045477	2466	821	93190.68	6.14	54.50	84.12	-0.137	Cytoplasm, Mitochondrion
ATG10	LOC125041822	675	224	26022.52	5.06	51.1	82.63	-0.485	Nucleus
ATG12	LOC125041823	363	120	13637.43	6.14	38.97	68.33	-0.718	Cytoplasm
ATG13	LOC125031250	1422	473	52171.50	5.40	49.86	73.21	-0.547	Cytoplasm, Nucleus
ATG14	LOC125035822	1467	488	54777.77	5.72	39.91	80.14	-0.510	Nucleus
VPS15	LOC125046127	3987	1328	148803.93	6.29	45.38	92.68	-0.176	Cytoplasm, Nucleus
ATG16	LOC125044282	1695	564	63011.29	6.93	44.3	85.5	-0.458	Nucleus
ATG17	LOC125042474	4143	1380	156986.09	5.14	51.27	79.86	-0.697	Nucleus
ATG18	LOC125034319	1035	344	36468.30	6.39	44.12	75.44	-0.190	Nucleus
ATG101	LOC125034760	660	219	25158.20	5.15	36.07	84.57	-0.415	Golgi apparatus, Nucleus

### Phylogenetic tree construction

**Fig. 1:**
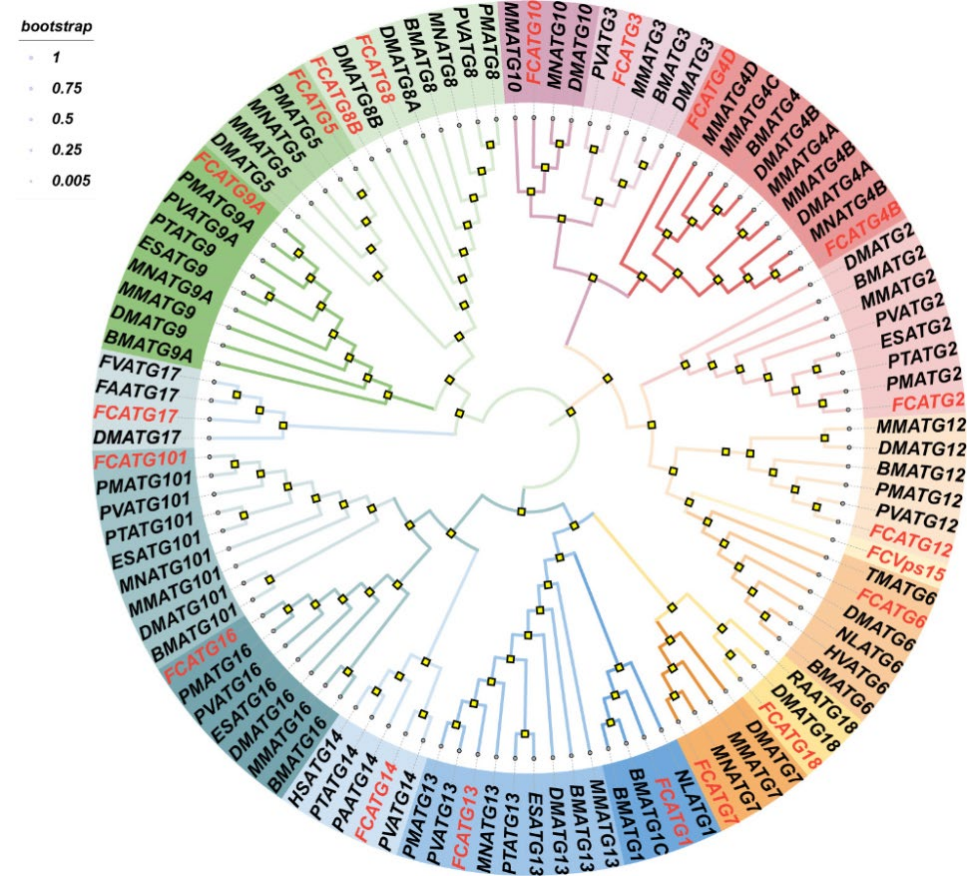
Phylogenetic relationships of the ATG genes *F. chinensis*

To explore the evolutionary relationships of *FcATG* genes, we constructed a phylogenetic tree using ATG protein sequences from fifteen species, including *F. chinensis*, *Mus musculus*, *Penaeus monodon*, *Litopenaeus vannamei*, *Macrobrachium nipponense*, *Bombyx mori*, and *Drosophila melanogaster* et al. (Fig. 1). The phylogenetic analysis revealed that the maximum likelihood (ML) tree for ATGs split into 18 subfamilies, with the subfamilies in *F. chinensis* clustering with their corresponding counterparts from other species as expected. The *FcATG*1, *FcATG*7, *FcATG*13, *FcATG*14, *FcATG*16, *FcATG*18, and *FcATG*101 subfamilies clustered first, followed by the *FcATG*2, *FcATG*3, *FcATG*4, *FcATG*6, *FcATG*10, *FcATG*12, and *FcATG*15 subfamilies, as well as the *FcATG*5, *FcATG*8, *FcATG*9, and *FcATG*12 subfamily. These results are consistent with the classification described in previous research and provide insights into the evolution of *FcATG*s orthologous genes in different species.

### Chromosomal distribution and structure analysis

**Fig. 2:**
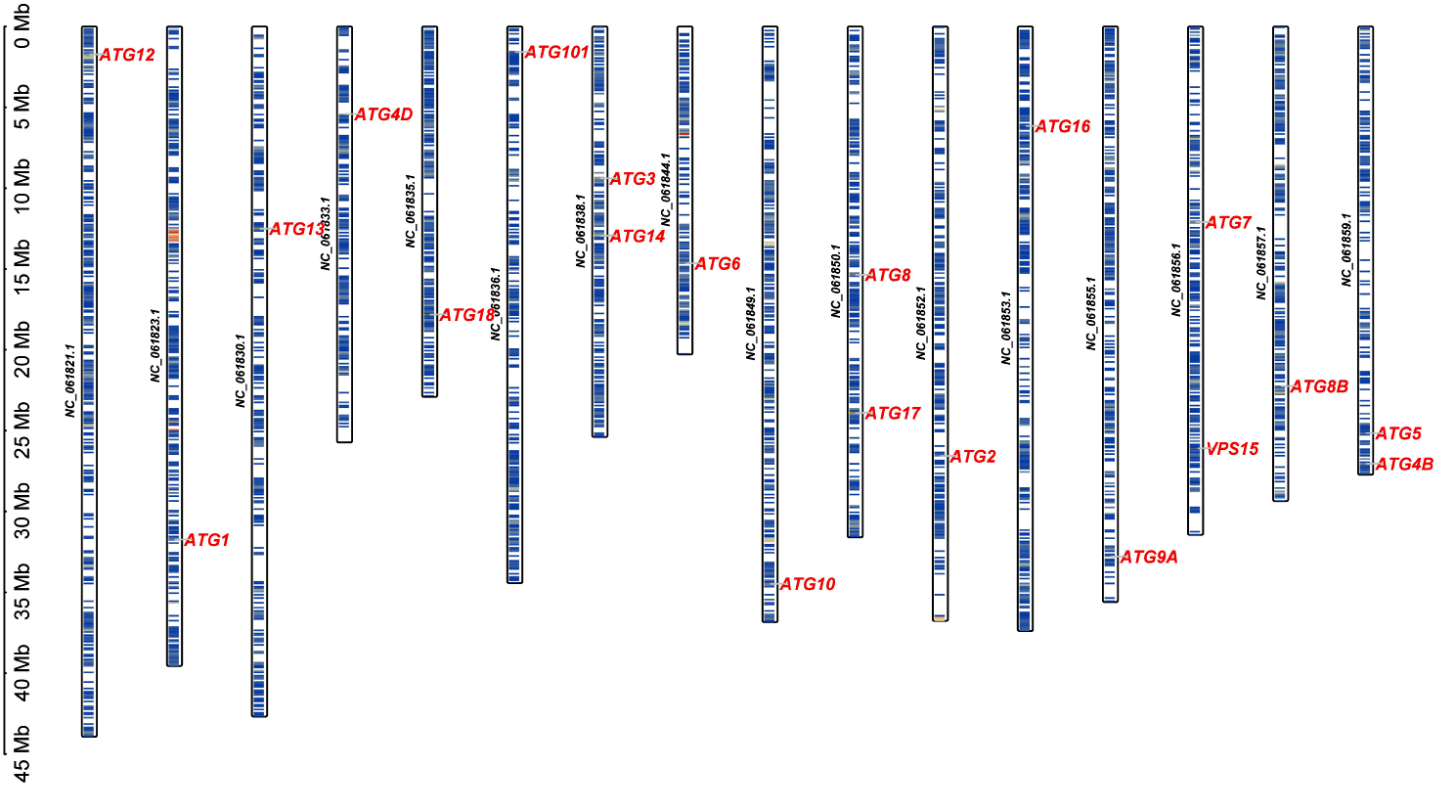
The chromosome location of ATG gene family members of *F. chinensis*

Chromosomal distribution analysis revealed that 20 ATG genes are distributed across 16 shrimp chromosomes, from NC-061821.1 to NC-061859.1. However, the distribution of ATG genes was uneven on each chromosome. For instance, *FcATG*3 and *FcATG*14, *FcATG*8 and *FcATG*17, as well as *FcATG*5 and *FcATG*4B are all located on the same chromosome, while the remaining chromosomes each hosted only a single ATG gene (Fig. 2). The non-random distribution of ATG genes in the shrimp genome highlights the importance of their genomic arrangement in autophagy-related processes.

**Fig. 3:**
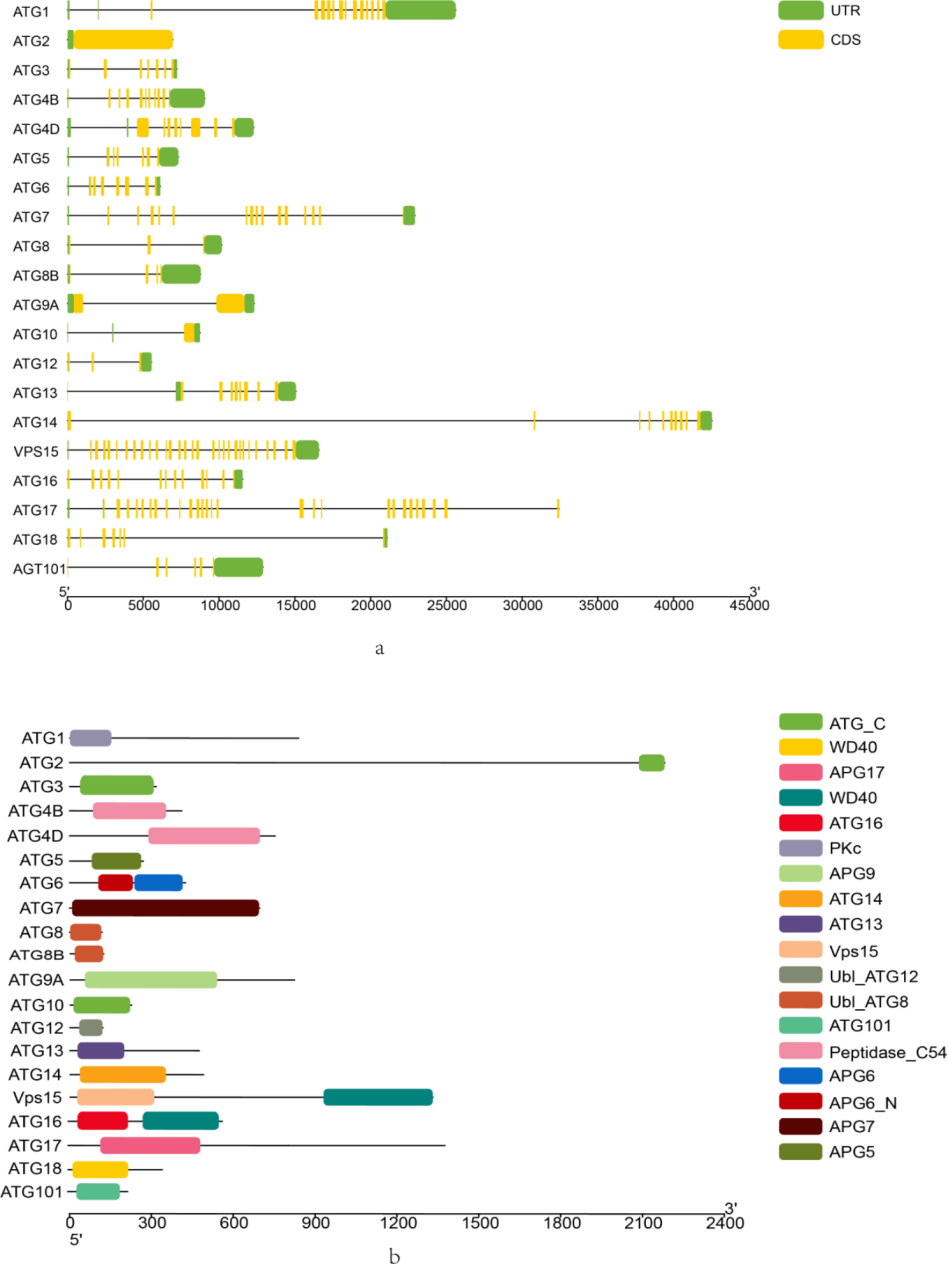
Schematic diagram of the gene structure (**a**) and protein domain (**b**) of ATG in *F. chinensis*

Based on the structural analysis of the *F. chinensis* ATG genes, the number of exons among ATG members varies from 1 to 29. Each ATG member comprises both UTR and CDS regions, with coding regions of similar lengths for members within the same subfamily, such as *FcATG*4B, *FcATG*4D, *FcATG*8, and *FcATG*8B (Fig. 3a). Protein domain analysis reveals that *FcATG*6, *FcVPS*15, and *FcATG*16 each possess two domains: APG6_N and APG6 for *FcATG*6, VPS15 and WD40 for *FcVPS*15, and *Fc*ATG16 and WD40 for *FcATG*16. In contrast, other proteins have only one domain. Different subfamilies exhibit distinct domain compositions. However, within the same subfamily, the domain structure is generally conserved. For example, both *FcATG*4B and *FcATG*4D contain the Peptidase_C54 domain, while *FcATG*8 and *FcATG*8B contain the Ubl_ATG8 domain (see Fig. 3b). This structural analysis offers insight into the diversity and conservation of gene architecture within the *FcATG* family and sheds light on potential functional implications.

### Expression profiles of ATG genes in *F. chinensis*

**Fig. 4:**
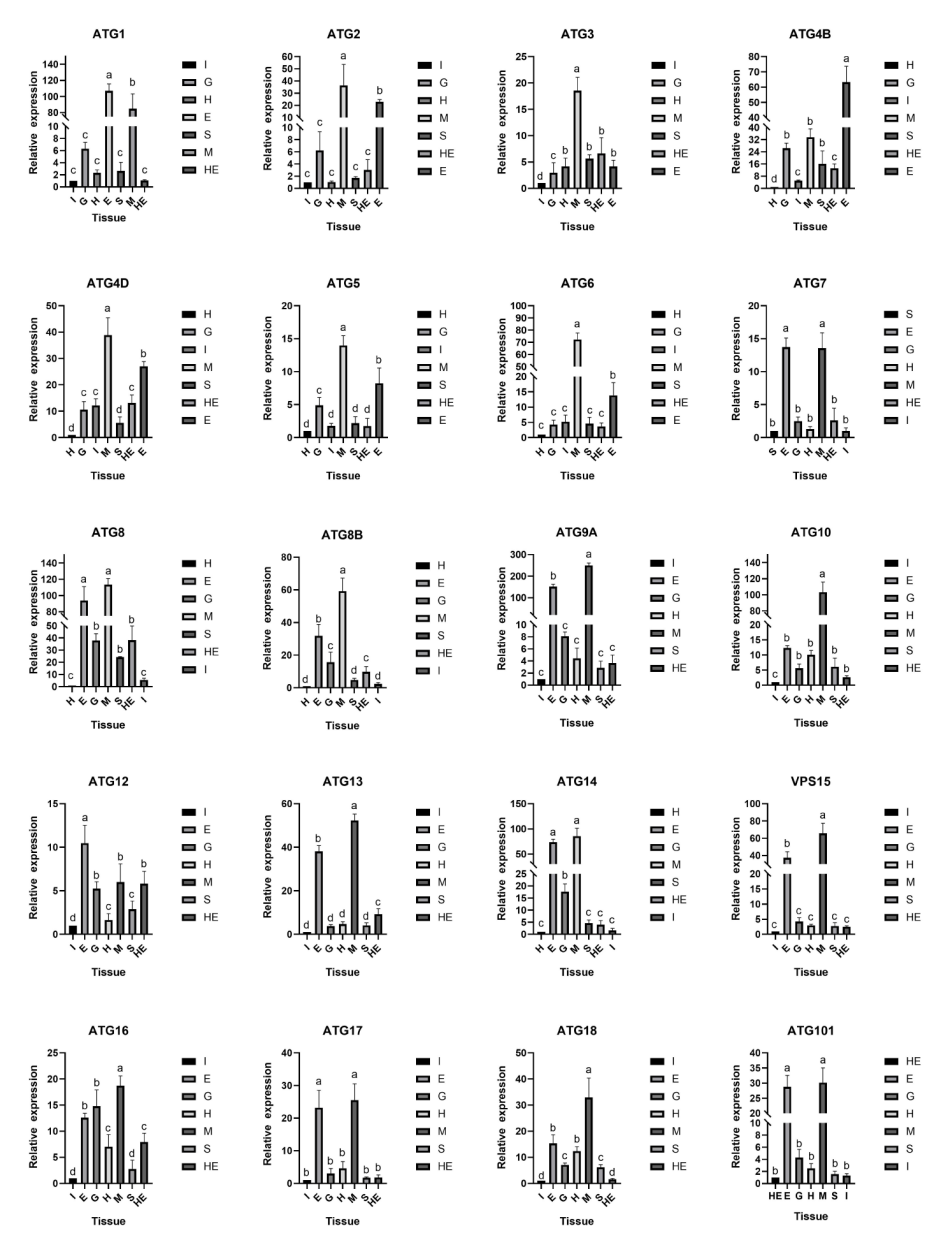
Expression levels of ATG in various tissues of healthy *F. chinensis*, including eyestalk (E), gill (G), heart (H), hepatopancreas (HE), intestine (I), muscle (M), and stomach (S)

The relative expression levels of *FcATG* were measured in various tissues, including the eyestalk, gill, heart, hepatopancreas, intestine, muscle, and stomach, using qRT-PCR with 18 S rDNA as the internal control in nine untreated shrimps. As shown in Fig. 4, *FcATG*1, *FcATG*4B, *FcATG*7, and *FcATG*12 genes exhibited higher expression levels in the eyestalk compared to the gills, heart, intestine, hepatopancreas, and stomach. The other 14 *FcATG* genes had the highest level of expression in muscles.

**Fig. 5:**
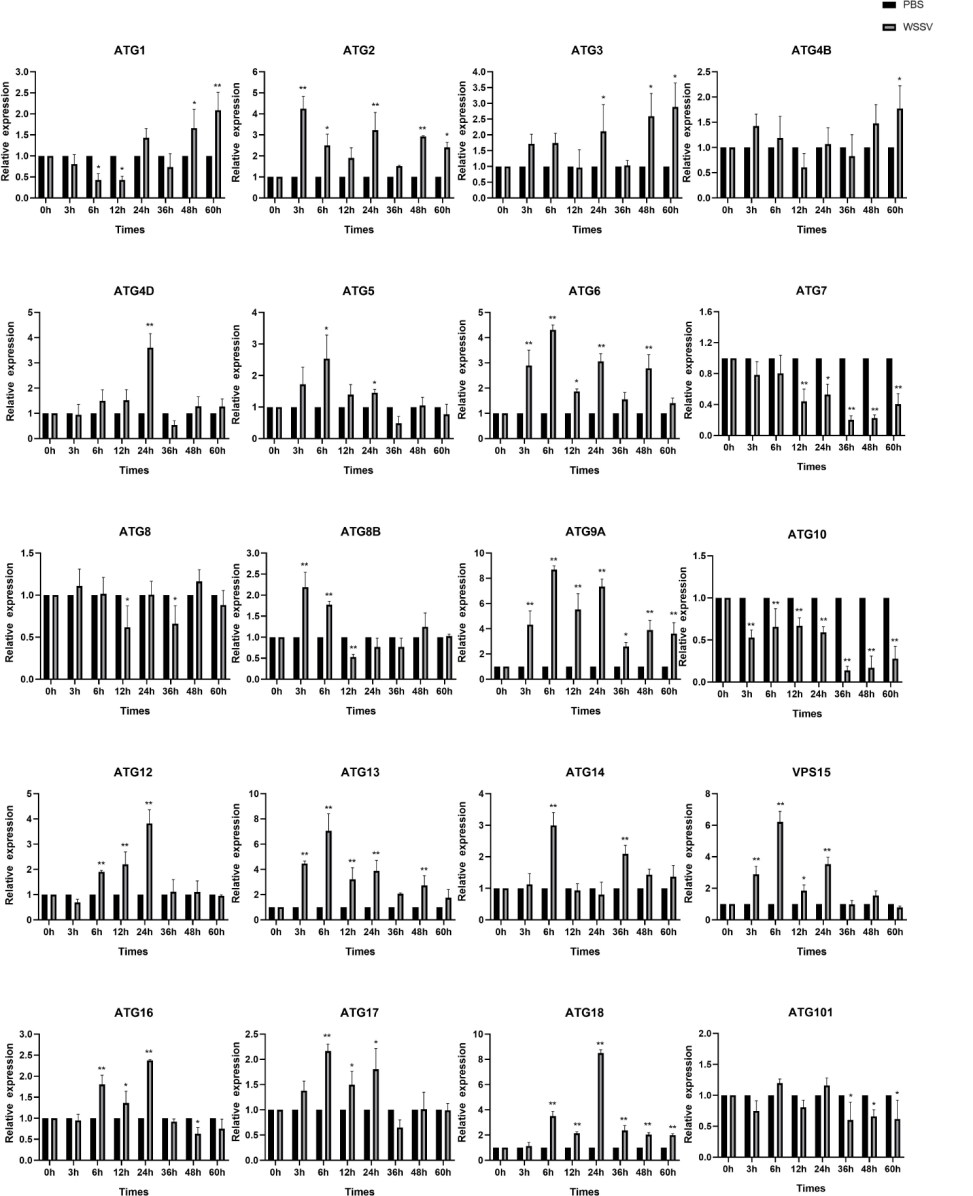
Expression patterns of 20 ATG genes in the hepatopancreas tissue of *F. chinensis* after WSSV infection. *Note* Error bars indicate the mean ± standard deviation (*n* = 3). * *P* < 0.05, ** *P* < 0.01, *** *P* < 0.001, and **** means *P* < 0.0001

### Expression profiles of ATG genes after WSSV infection

This experiment utilized qRT-PCR analysis to study the expression levels of the 20 identified members of the *FcATG* gene family. The cDNA samples from the hepatopancreas were collected at 3 h, 6 h, 12 h, 24 h, 36 h, 48 h, and 60 h after WSSV injection to investigate the expression levels of each member during WSSV infection. Figure 5 shows that *FcATG*1, *FcATG*3, and *FcATG*4B had a significant upregulation trend, with the highest expression at 60 h. *FcATG*7 and *FcATG*10 exhibited a noticeable downregulation trend. Most ATG genes showed an up-regulation followed by a down-regulation trend, with *FcATG*2, *FcATG*8, and *FcATG*8B peaked at 3 h. *FcATG*5, *FcATG*6, *FcATG*9A, *FcATG*13, *FcATG*14, *FcVPS*15, *FcATG*17, and *FcATG*101 reached their peak expression at 6 h. *FcATG*4D, *FcATG*12, *FcATG*16, and *FcATG*18 reached their highest expression at 24 h.

### Expression profiles of ATG genes after low-salt stress

**Fig. 6:**
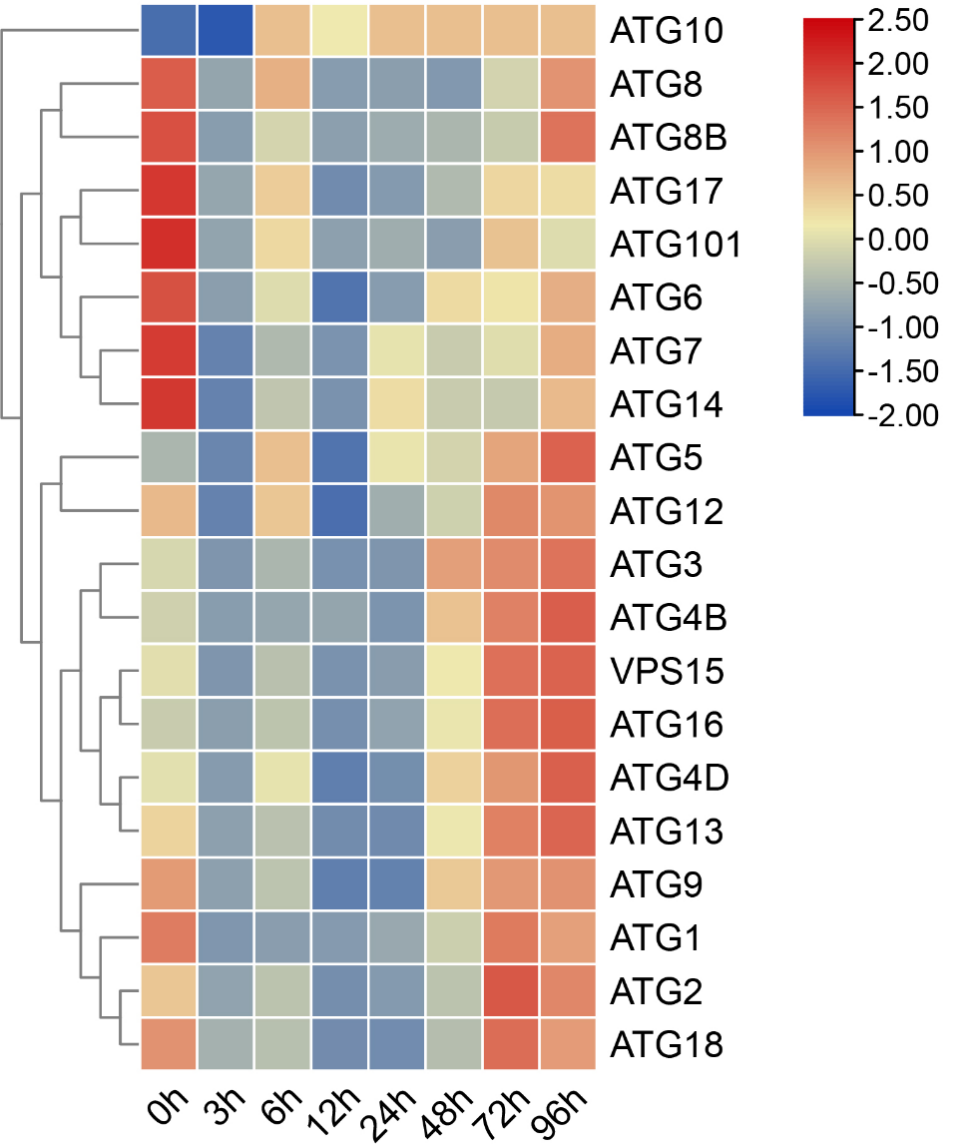
Heatmap of ATG genes expression in *F. chinensis* following experimental with low-salt stress from hepatopancreas tissues

The expression of *FcATG* genes in hepatopancreas tissues after low-salt stress was analyzed using qRT-PCR (Fig. 6). The results showed an up-regulation trend for *FcATG*1-3, *FcATG*4B, *FcATG*4D, *FcATG*5, *FcATG*9, *FcATG*12-13 *FcVPS*15, *FcATG*16, and *FcATG*8, peaking at 72–96 h. *FcATG*10 exhibited a notable upregulation, reaching peak expression at 24 h. Meanwhile, *FcATG*6- *FcATG*8, *FcATG*8B, *FcATG*14, *FcATG*17, *FcATG*101 initially showed a downregulation followed by an upregulation trend. *FcATG*17, and *FcATG*101 peaked at 72 h, while the rest reached their highest expression at 96 h.

### Protein interaction analysis

**Fig. 7:**
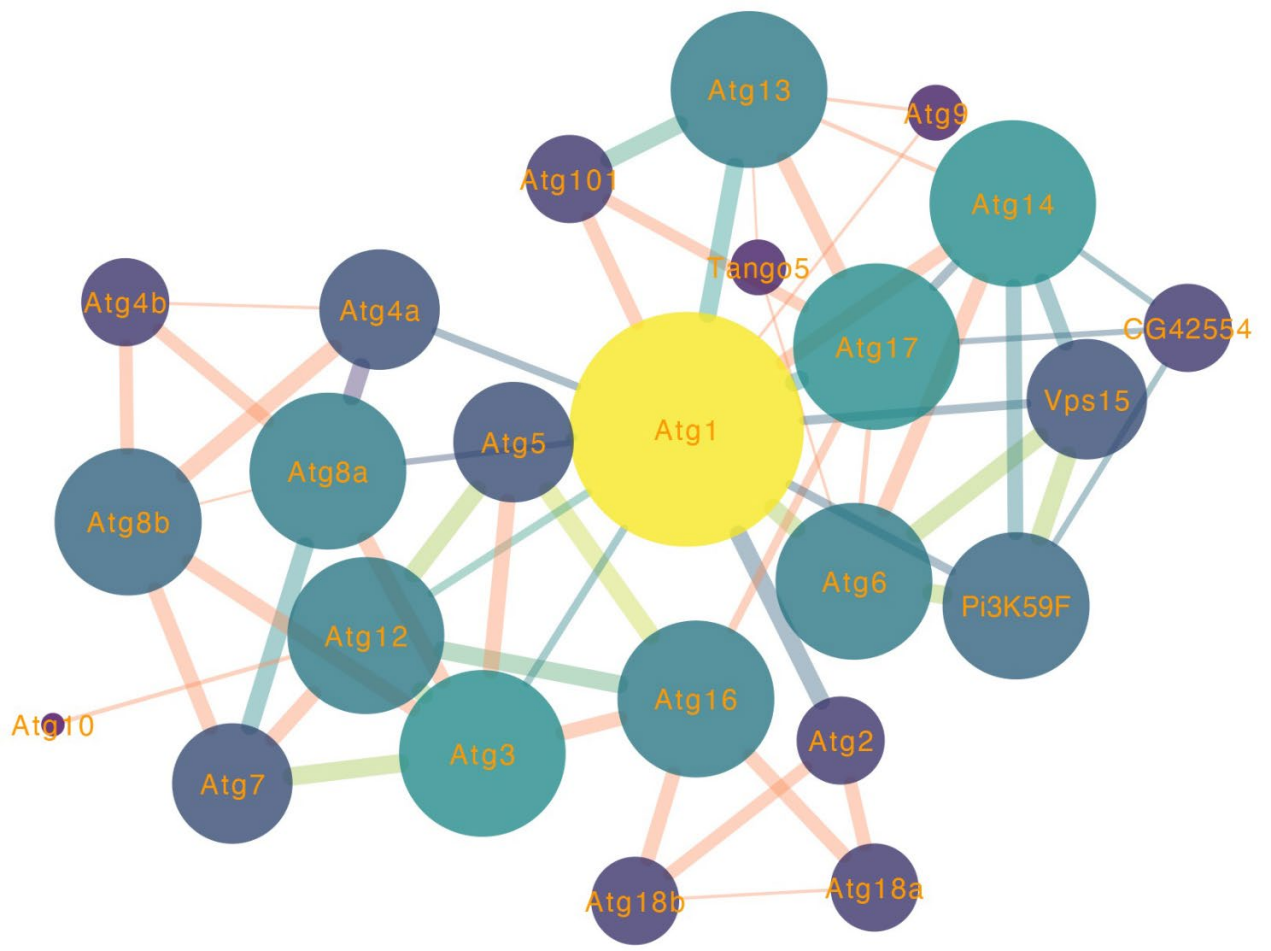
Predicted functional partners of the ATG genes in *F. chinensis* using the protein–protein interactions (PPIs) method

To explore the intricate interaction networks of ATGs in *F. chinensis*, putative interaction networks were constructed using the online STRING server based on orthologous genes in *D. melanogaster* (Fig. 7). The results revealed that within the ATG gene family, 20 ATG proteins displayed mutual interactions. Among these, proteins such as Pi3K59F, Tango5 and CG42554 exhibited high connectivity to multiple ATG genes, indicating a close predicted interaction with ATG proteins in *F. chinensis*.

## Discussion

Autophagy is a highly conserved catabolic pathway that is involved in the cellular degradation of long-lived proteins or dysfunctional cellular components through lysosomes action. It is considered a pro-survival mechanism and for maintaining homeostasis [21]. Meanwhile, autophagy is a sensitive process that underlies cell response to almost every stressful condition affecting cellular homeostasis. Its role is complex and likely depends on the cell’s genetic background and environmental cues [22, 23]. Recent research has shown that autophagy is crucial for maintaining homeostasis at both the cellular and organismal levels [24–26]. While the study of ATG genes has been characterized in yeasts and mammals for decades, there has been a lack of systematic identification and analysis of the ATG gene family in *F. chinensis*. Additionally, research on ATGs under biotic and abiotic stresses of *F. chinensis* has not been conducted. This study identified and characterized the ATG genes of *F. chinensis*, including their phylogenetic analysis, protein structures, and physicochemical properties of ATGs. In addition, the expression profiles of ATG genes were analyzed after WSSV infection, low salt stress, and in healthy tissues. These analyses provide insights into the involvement of ATGs in response to toxicological and environmental stresses.

This study reports the identification of 20 ATG family members in the *F. chinensis* genome, three of which have instability coefficients below 40, indicating relative stability of the gene family. This finding is consistent with the results reported by Liu et al. [27] in their investigation of the physical and chemical properties of the ATG gene in *Punica granatum*. Meanwhile, the 20 ATG gene family members of *F. chinensis* are distributed unevenly across 16 chromosomes. This distribution pattern is similar to that found in *Arabidopsis* [28], suggesting that even within the same family, members can have different chromosomal positions. The ATG genes are relatively conserved from yeast to humans, but the detailed evolutionary history remains unclear. Analyzing phylogenetic relationships can provide insight into the evolution of the ATG genes. A phylogenetic tree was constructed based on the amino acid sequences of ATG homologous genes from 15 different species. The resulting tree revealed that 20 shrimp ATG genes were grouped into 18 subfamilies. Moreover, the evolutionary relationship between *L. vannamei* and the ATG genes of the Chinese shrimp ATG family was found to be similar, suggesting a close association. As per Zhu’s findings, the ATG gene of *Procambarus clarkii* and *Homarus americanus* are closely related [26]. In general, proteins within the same subfamily on a branch exhibit similar structures and functional domains, thereby indicating a tendency towards functional conservation.

Previous studies have profiled the expression patterns of ATG family members in crustacean [20, 29–31]. However, such research has not been conducted in *F. chinensis*. To better understand the characteristics and functions of ATG genes in *F. chinensis*, we used qRT-PCR to profile the expression patterns of ATG genes in different tissues. ATGs are expressed ubiquitously in various *F. chinensis* tissues with varying levels, indicating their divergent functions in the organism. Sixteen ATG genes have the highest expression levels in muscles, while four ATG genes have the highest expression levels in the eye stalk, suggesting that ATG genes may be involved in the growth and development process. These finding are consistent with the research results in *P. monodon*, *L. vannamei* and *Ctenopharyngodon idellus* [20, 31, 32]. Intriguingly, the expression level of *FcATG*16 was notably higher in the gill compared to other ATG family members. The gill is a multipurpose organ that provides gas exchange and osmotic regulation. This suggests that *FcATG*16 may play an important role in the physiological function of the gill [20, 30]. Besides, ATG3 is highly expressed in hepatopancreas, which is an important immune organ, suggesting ATG3 may be involved in immune responses, lipid metabolism, and detoxification in *F. chinensis*, as previous studies have demonstrated in other species such as *Pelteobagrus fulvidraco*, *Macrobrachium rosenbergii*, and some mammals [33–36]. Taken together, ATG genes may participate in various biological processes, with some members specifically related to the immune system of *F. chinensis*.

Studies have shown that the expression of ATGs can be affected by various stimuli, such as environmental stress and pathogen invasion, and these genes play important roles in mediating autophagy, participating in embryonic development, host resistance to viral and bacterial infection, and immunity process [37, 38]. Recently, there have been several efforts to study the role of the ATG gene family in pathogen invasion. The results have shown that different ATG members are stimulated after virus infection in various species. For instance, in olive flounder *Paralichthys olivaceus*, the mRNA level of ATG6 significantly increased after viral hemorrhagic septicemia virus (VHSV) infection [39]. In *Procambarus clarkii*, ATG14 expression was initially upregulated upon WSSV infection and then stabilized [26]. Due to the limited information on ATG in crustaceans, only the expression patterns of individual members in response to virus invasion were detected. Therefore, we conducted WSSV infection experiments and measured the expression levels of all *FcATG* members in *F. chinensis*. The results showed that in the hepatopancreas, 12 *FcATG* genes exhibited an initial up-regulation followed by a down-regulation trend. Among them, ATG6, ATG9A, and ATG14 genes underwent significant changes, with peak expression levels occurring at either 6–24 h post-treatment. This is consistent with the changes in the ATG6 gene observed in *P. vannamei*’s responds to WSSV infection [20], suggesting that *FcATG6* and *FcATG14* play an crucial role in *F. chinensis*’s response to biotic stress. Unlike other ATG family members, the expression levels of three genes (*FcATG*1, *FcATG*3, and *FcATG*4B) gradually increased until 60 h after injection. This indicated that these genes can be significantly induced by WSSV infection. It is believed that autophagy-related genes may be regulated thus cellular autophagy be affected in the early stage of viral infection. In the late stage of infection, host cells downregulate the expression of ATG to resist the virus and prevent its proliferation [40]. In contrast, the expression trend of *FcATG*7 and *FcATG*10 was continuously down-regulated after WSSV infection. This suggests that these genes may have species-specific or environment-dependent effects on the response of *F. chinensis* to WSSV infection. On the other hand, the expression trends of *FcATG*8 and *FcATG*101 did not change significantly. It is speculated that they may not be the main genes involved in coping with WSSV in *F. chinensis*, and further research is need to confirm this hypothesis. In summary, the results of *F. chinensis* suggest that ATGs play a crucial rule in the immune response against WSSV, emphasizing their significance in immunity.

Further, autophagy is essential for maintaining the intracellular stability under normal and stress conditions and it ensures quality control within cells. However, autophagy induced by ion concentrations, pH, and other stressors, particularly when the stress level is not fatal, constitutes a strategy to adapt and cope with stress. This can promote cell survival by maintaining a sufficient amino acid pool and cell energy level [41, 42]. For example, *L. vannamei* and *Macrobrachium japonicum* are able to regulate autophagy in response to abiotic stresses such as low temperature and hypoxia [31, 43]. In this study, 12 *FcATG* genes were significantly induced, and their transcript abundance peaked at 96 h after treatment under low-salt conditions. The expression levels of *FcATG5* and *FcATG12* were significantly upregulated, suggesting that these *FcATG* genes are activated to promote cell survival by regulating autophagy when challenged by low-salt conditions. Similarly, the expression levels of ATG5 and ATG12 were also significantly upregulated under pH or carbonate alkalinity stress [44], indicating that the *FcATG5* and *FcATG12* genes may play an crucial role in the adaptation of *F. chinensis* to abiotic stress. Previous studies have shown that *F. chinensis* increases body energy consumption and enhances sugar metabolism activity in response to abiotic stress, including the acute changes in salinity [45]. Autophagy is proposed to participate in the process of sugar metabolism by regulating glucose uptake, key enzymes of glycolysis, and mitochondria [46]. Taken together, these results demonstrate that abiotic stress alters the expression of ATG genes in organisms and plays an important role in their response to such stress.

In addition, the connectivity of ATG genes in *F. chinensis* was firstly analyzed via PPI network analysis to further investigate the regulatory mechanisms of these ATG genes. The ATG genes exhibited high connectivity with genes related to the PI3K pathway, which has now been demonstrated to be involved in numerous important physiological processes and immune responses in aquatic animals. PI3Ks can regulate NK cytotoxic activity and inhibit NADPH oxidase [47]. Following a Vibrio infection, the expression level of PI3K in *Haliotis disversicolor* was found to be significantly elevated in various tissues [48]. Similarly, *Scophthalmus maximus* employed PI3K signaling pathway to regulate ion transport in response to low-salt stress [49]. According to our previous experiments, the induced upregulation of PI3K-related genes responded to high alkali and elevated pH stresses by activating the PI3K pathway in *F. chinensis*. Therefore, we hypothesize that the ATG genes’s response to WSSV injection and low-salt stress may also be mediated through the PI3K pathway in *F. chinensis*. This study will enhance our understanding of the molecular mechanisms underlying how *FcATGs* respond to various environmental stressors.

## Conclusions

In summary, we identified 20 ATG genes in *F. chinensis*, and characterized their structure, domains, phylogenetic tree, chromosomal distribution, and bioinformatics information. The qPCR examination results indicated that ATG genes played an essential role in WSSV infection. Furthermore, we analyzed their expression profiles based on generated from low-salt stress, providing potential functional information on the response of ATG genes to abiotic stressors in *F. chinensis*. This study enhances our comprehension of the molecular basis of the ATG gene family’s response to toxicological and environmental stresses in *F. chinensis.* It provides important clues for future research on their functions.

## Materials and methods

### Experimental Shrimp

In the present study, *F. chinensis* (weight 15.4 ± 2.1 g, length 11.1 ± 0.87 cm) were obtained from the Changyi Haifeng Aquaculture Co., Ltd., Shandong, China. The shrimps were cultured in cement breeding ponds, with continuous aeration, at a temperature of 25 ℃, salinity of 30, and a pH of 8.4. During the acclimation period, the shrimps were fed thrice daily at 00:00, 09:00, and17:00 for two weeks. After acclimation, nine healthy and energetic *F. chinensis* were randomly selected for the study. Seven tissues, including eye stalks, gills, heart, hepatopancreas, muscles, stomach, and intestines, were collected and stored in liquid nitrogen for later use.

### Identification of ATG gene family in *F. chinensis*

The ATG gene sequences of humans, mouse, zebrafish and arthropods were obtained from the UniProt (https://www.uniprot.org/) and NCBI (http://www.ncbi.nlm.nih.gov/) databases, and were used as the query sequences to search against the whole genome databases of *F. chinensis* using the TBLASTN alignment tool (E-value = 1e^− 5^). The amino acid sequences were predicted and translated from the open reading frame (ORF) using ORF Finder (https://www.ncbi.nlm.nih.gov/orffinder/). The results were validated by BLASTP against the NCBI non-redundant protein (NR) database. The domain architectures of the ATG in *F. chinensis* were examined using SMART to confirm all of the family genes. The functional interaction network of ATG genes in *F. chinensis* was investigated by utilizing the STRING online server (https://cn.string-db.org/) based on orthologous genes identified in *D. melanogaster*. The resulting network was then visualized using Cytoscape v 3.7.

### Phylogenetic analysis

The amino acid sequences of ATG subunits from *F. chinensis* and several representative animals were obtained from NCBI and aligned using the ClustalW program. Phylogenetic trees were constructed using the Maximum Likelihood (ML) method with a bootstrap value of 1000 in MEGA 11. The resulting tree was annotated using iTOL (https://itol.embl.de/login.cgi).

### Bioinformatics analysis

To analyse the characteristics of ATG, we predicted the amino acids, molecular weight (MW), theoretical isoelectric points (pI), instability index, and grand average of hydropathicity (GRAVY) of the ATG genes using ExPASy ProtParam (https://web.expasy.org/protparam/). We used SignalP 5.0 [50] to predict signal peptides and WOLF PSORT [51] to predict subcellular localization. NCBI-CDD (https://www.ncbi.nlm.nih.gov/Structure/bwrpsb/bwrpsb.cgi) was used to predict the conserved domain of ATG genes. The structures of ATG genes were visualized using TBtools.

### The WSSV infection experiment

Preparation of WSSV stock solution: the carapace and hepatopancreas were removed from WSSV - infected shrimp before shearing in an ice - bath environment, and 1 g tissue was collected and mixed with 10 ml phosphate - buffered saline (PBS). The mixture was homogenized at 10,000 rpm for 5 s and then centrifuged at 8000 rpm for 5 min at 4 °C. The supernatant fluid was filtered through a 0.45 μm microporous membrane as a virus stock solution [52]. 10^3^-diluted-stock-solution (using pre-chilled PBS) was used in this experiment to infect healthy shrimps. Shrimps sampled from the shrimp ponds were verified to be WSSV-free after being tested for related pathogens before experimentation.

After acclimation, a total of 180 healthy *F. chinensis* were selected for the WSSV infection experiment. The shrimps were randomly divided into two groups, with three replicates in each group and 30 shrimps in each replicate. The second abdominal segment muscle of the shrimps was injected with PBS or WSSV suspensions. The experimental group received 10 µL of WSSV virus suspension, while the control group was injected with 10 µL sterile phosphate (PBS). The injected shrimps were raised separately in aquariums and all showed symptoms of WSSV infection. We randomly selected hepatopancreas tissues from three healthy and complete shrimps were randomly selected at 0, 3, 6, 12, 24, 36, 48, and 60 h after injection and frozen immediately in liquid nitrogen and then stored at − 80 ℃.

### The low salinity stress experiment

Ninety healthy and energetic shrimps were randomly selected and divided into three groups, each containing 30 shrimps. The stress treatment group was subjected to low-salt stress conditions with a salinity of 15. Salinity was corrected every 6 h using with light brine during the experiment. The hepatopancreas tissues were collected after exposure to low-salinity stress at 0, 3, 6, 12, 24, 48, 72, and 96 h. Three parallel samples were taken at each time point, frozen in liquid nitrogen and then stored at − 80 ℃ for later use.

### Total RNA extraction, and cDNA synthesis

The total RNA of tissues was extracted using TransZol Up (TransGen Biotech, China) following the manufacturer’s instructions. RNA concentration and integrity were measured using an Ultra-trace UV spectrophotometer (Thermo, USA) and 1.5% agarose gel electrophoresis (AGE). According to the manufacturer’s instructions, the first-strand cDNA synthesis was performed with HiScript III RT SuperMix for qPCR (+ gDNA wiper) (Vazyme, China).

### Quantitative real-time PCR (qRT-PCR) analysis

The expressions of ATGs in different tissues of healthy *F. chinensis*, as well as their temporal expression patterns in the hepatopancreas after WSSV infection and low-salt stress were detected by qRT - PCR. Primers were designed for each gene using Primer5 software, avoiding regions with hairpin structures as identified by Mfold at 60 °C [53]. The 18 S rRNA was selected as the reference gene. Primer specificity and efficiency were checked for all qPCR conditions. The primer sequences are listed in Table 2. The qRT-PCR assay was conducted using ChamQ SYBR Color qPCR master mix (Vazyme, China) in a final volume of 10 µl on the FAST 7500 Real-time system (FAST, USA). The reaction mixture contained ChamQ SYBR Color qPCR Master Mix (Low ROX Premixed) (Vazyme, China), forward and reverse primers (final concentration 100 nM) and 4 µl of diluted cDNA. The PCR reactions were initiated with a denaturation step at 95 °C for 30 s, followed by 40 cycles of two-step amplification as follows: denaturation at 95 °C for 10 s, annealing at 60 °C for 30 s. Fluorescence data were acquired during the final step. Gene-specific amplification was confirmed by a single peak in the melting curve analysis. Three technical repeats were performed for each biological repeat. The data was analysed using FAST 7500 software, and the relative expression ratio was calculated using 2^−ΔΔCT^ method [54]. Statistical analysis was performed using SPSS 19.0. A One-Way ANOVA was exerted to compare the differences between groups. Finally, GraphPad Prism was used for scientific graphing.

**Table 2:** The primers used in this research

Primer	Sequence (5’-3’)	Purpose
*FcATG1-F*	CTTATGGAGCGAGAGCACAATGAG	PCR
*FcATG1-R*	GAGAGCGAGCCAATTCAAGGATAC	PCR
*FcATG2-F*	AGCAAGTTGGTAGGAGGTGTCAG	PCR
*FcATG2-R*	TGAGCCGATGAAGTTGTGAATGC	PCR
*FcATG3-F*	CGCCGAGTATCTTACGCCAATTC	PCR
*FcATG3-R*	GGACAGTGATGAACCAAGTGATCTC	PCR
*FcATG4B-F*	CAGCCTTGCGGAGTGATGTG	PCR
*FcATG4B-R*	CAGCCCTTGTCAGATGTAAAGTTTG	PCR
*FcATG4D-F*	TGGAGGCATGAGCGTGTCTG	PCR
*FcATG4D-R*	GGCACTGGGTCTACTGGGATG	PCR
*FcATG5-F*	ATGATGCTGAGATGTGGTTGGAATC	PCR
*FcATG5-R*	GTGGGAGAAGTGGGCTGTGAG	PCR
*FcATG6-F*	CACCATCAACAACCTCCGCTTAG	PCR
*FcATG6-R*	CGGTGCTTGGAGAACTTCAGTC	PCR
*FcATG7-F*	AGCAACCAGCCAGTACCTCAC	PCR
*FcATG7-R*	AGACGCTCCAGCAGACTTCAG	PCR
*FcATG8-F*	GGGCAGAGGGAGAGAAGATTAGG	PCR
*FcATG8-R*	TTGTCAAGATCGCCGATTCGTG	PCR
*FcATG8B-F*	AGGAGTTTCGCCCAGAGACAG	PCR
*FcATG8B-R*	ATACCGCTCAATAATCACAGGAACC	PCR
*FcATG9A-F*	CACGCCGCTCATTCTCATCTTC	PCR
*FcATG9A-R*	CCCACGCCTACAACCTCTACG	PCR
*FcATG10-F*	CTGACGCAGCAAGAGCATCC	PCR
*FcATG10-R*	AACCAGCTTATCATGTACCTTAGGC	PCR
*FcATG12-F*	AGAATAAACACACAGCCGCCAAG	PCR
*FcATG12-R*	GTACCTGCGTATGAATTCTGCTACC	PCR
*FcATG13-F*	CAGCAACAGCAACAGCAACAAC	PCR
*FcATG13-R*	AGGGAATATCGGGCAGGAAGAAG	PCR
*FcATG14-F*	AGACACAGTATCGCATTGTTCACC	PCR
*FcATG14-R*	TTCTCCATCACTCTCCTCACACTC	PCR
*FcVPS15-F*	CGGCAGTGGTGGCGAGAG	PCR
*FcVPS15-R*	GCAGGCATGAGAGGATACATACTTC	PCR
*FcATG16-F*	TTAACTTAGCCTTCACTGCCTTGG	PCR
*FcATG16-R*	TGTGTCATTCTCCAGGTTCAACTTC	PCR
*FcATG17-F*	CATTGACTATGGCTCCGACACTG	PCR
*FcATG17-R*	GCTGCTGGCATATTCTCGTCTG	PCR
*FcATG18-F*	TTCCTCATCGCCTCCTCCAATAC	PCR
*FcATG18-R*	GGCTGCTCCTCTACCACTACTC	PCR
*FcATG101-F*	AAGGCAGGCAGGTGGATGAG	PCR
*FcATG101-R*	CTATGGTTCCAACGGCGTATGTG	PCR
*Fc18S-F*	TATACGCTAGTGGAGCTGGAA	PCR
*Fc18S-R*	GGGGAGGTAGTGACGAAAAAT	PCR

## Data Availability

The sequence information of *Fenneropenaeus chinensis* ATG family genes were collected from *Fenneropenaeus chinensis* genome to Breeding Database (https://www.ncbi.nlm.nih.gov/datasets/genome/GCF_019202785.1/), and the ATG protein sequences of *Mus musculus*, *Litopenaeus vannamei*, *Macrobrachium nipponense*, *Bombyx mori*, and *Drosophila melanogaster* et al. were downloaded from the NCBI website (https://www.ncbi.nlm.nih.gov/). All data used during the current study are included in this published article and its supplementary information files or available from the corresponding author on reasonable request.
